# A Review of the Capacitive MEMS for Seismology

**DOI:** 10.3390/s19143093

**Published:** 2019-07-12

**Authors:** Antonino D’Alessandro, Salvatore Scudero, Giovanni Vitale

**Affiliations:** Istituto Nazionale di Geofisica e Vulcanologia, Osservatorio Nazionale Terremoti, 00143 Rome, Italy

**Keywords:** MEMS, capacitive accelerometer, seismology, earthquake

## Abstract

MEMS (Micro Electro-Mechanical Systems) sensors enable a vast range of applications: among others, the use of MEMS accelerometers for seismology related applications has been emerging considerably in the last decade. In this paper, we provide a comprehensive review of the capacitive MEMS accelerometers: from the physical functioning principles, to the details of the technical precautions, and to the manufacturing procedures. We introduce the applications within seismology and earth sciences related disciplines, namely: earthquake observation and seismological studies, seismic surveying and imaging, structural health monitoring of buildings. Moreover, we describe how the use of the miniaturized technologies is revolutionizing these fields and we present some cutting edge applications that, in the very last years, are taking advantage from the use of MEMS sensors, such as rotational seismology and gravity measurements. In a ten-year outlook, the capability of MEMS sensors will certainly improve through the optimization of existing technologies, the development of new materials, and the implementation of innovative production processes. In particular, the next generation of MEMS seismometers could be capable of reaching a noise floor under the lower seismic noise (few tenths of ng/$\sqrt{\mathrm{Hz}}$) and expanding the bandwidth towards lower frequencies (∼0.01 Hz).

## 1. Introduction

Progresses in seismology firmly rely on the development of instrumentation. The first, although pioneering tools, were only used to display the ground shaking after the strongest earthquakes. The technical advancements throughout the 20th century made it possible to realize increasingly reliable and sensitive seismometers thus that today, seismometers are able to characterize the ground motion with great accuracy and over a wide range of frequency. From a scientific point of view, accuracy is crucial: the greater is the quality of the observation, the more reliable will be the knowledge of the earthquake-related phenomena and, in turn, the following considerations about seismic hazards.

Further developments in the next future will likely improve even more the present-day capabilities, not without a tendency for the price of professional instruments to rise exponentially. For this reason, at least for some applications, the trend is a compromise between the quality of the sensor and the quality of the results, which, in the last few decades, resulted especially in the technological development of miniaturized systems. Traditional sensors based on a spring-mass principle have heavy proof masses making them bulky and difficult to transport and manage. Miniaturized sensors, on the other hand, reduced the size and weight, in addition to the shock survivability and the reduced cost. The application of Micro Electro-Mechanical Systems (MEMS) to seismology (and related branches) is very recent: in the book “Instrumentation in Earthquake Seismology” edited in 2004 [1], the MEMS were not even mentioned. The second edition, published twelve years later [2], introduced some new trends in seismic sensors, and, among others, a comprehensive section on micromachined devices.

**Figure 1:**
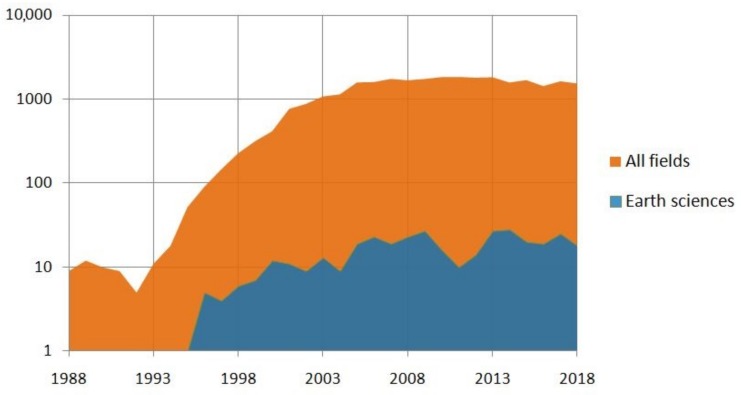
Number of scientific papers containing the term “MEMS” in the title from Scopus database (accessed on February 2019).

The term “MEMS” in academic literature appears since the mid 1980s. The database Scopus returned more than 29,000 entries with the word “MEMS” in the title in February 2019; among those, only the 0.6% is listed in the field of Earth Sciences (which includes seismology and geophysics), while the most cited discipline is Engineering (>40%). The graph in Figure 1 shows the number of indexed papers in which the term MEMS is contained in the title in the last 30 years. Since the early 1990s to the early 2000s, the topic grew exponentially, while in the last ten years the trend is constant, with ca. 1700 papers on the average, and ca. 20 papers in the field of Earth Sciences.

Since 1979, the Stanford University (USA) hosted the first pioneering demonstrations of the use of MEMS accelerometers [3,4], long before they became the extremely popular products they are today. MEMS accelerometers are today used in a multitude of fields and are ideal for large-scale and large-volume applications (e.g., monitoring of machines and vehicles, navigation systems, etc.) [5,6,7,8]. Later, boosted by space exploration necessities, sensors became even more lightweight and sensitive, and suitable also for the requirements of earthquake monitoring [9,10]. The increasing capillarity of their diffusion is literally revolutionizing both the industry and the market. A comprehensive list of the variety of possible applications of MEMS devices is given in [8,11]. Schematically, a MEMS device can be defined as a system capable of sensing the external environment and then convert the measured physical quantities into electrical impulses. Therefore, a MEMS is a system capable of getting information from the external environment converting the measured physical quantities into electrical impulses. MEMS are fabricated directly upon a silicon substrate and can reach the dimension of a few microns. MEMS accelerometers can be classified according to the physical effect used in the sensing mechanism: capacitive, piezoresistive, piezoelectric, electrochemical, optical, thermal, etc. Each method has its own advantages and disadvantages. For example, piezoelectric accelerometers are not suitable for static acceleration, while the capacitive ones are generally more performing and reliable.

This review aims to provide the state-of-the-art of the use of MEMS in the broad field of seismology after about 10 years from the first significant applications. At present, such contribution is missing, with only a short review recently proposed [12]. We describe the functioning principles of the capacitive MEMS which are the most suitable for applications within the sensing and the frequency ranges of seismological interest. We introduce the basics of the capacitive devices, treating all the details about the various systems’ configuration, the constructive processes, and the precautions on their use as seismic sensors. We present the present-day applications and give an outlook for the possible future ones.

## 2. The Capacitive MEMS Accelerometers

Accelerometers are inertial sensors designed to detect velocity variations, typically in the medium-to-high range of frequency. Among the various typologies of MEMS accelerometers, the electromechanical capacitive sensors dominate the market and are widely chosen for many applications. The highest resolution is in the high frequency range: typically from hundreds of Hz to some *k*Hz, but, using the electronic capacitance-switch, they can also respond to lower frequencies and continuous components.

An accelerometer is a directional device; therefore, it is able to detect accelerations along a single axis. To measure accelerations on the three dimensions, three accelerometers, oriented along the axes of an orthogonal triad, are then necessary. This task could be challenging in the microscopic scale where the flat base of silicon wafer sets the manufacturing limits. If it could be relatively easy to manufacture two identical structures on the plane, it is harder to extend the structure perpendicularly off of plane. For this constructional reason, the type of electrodes employed for the manufacture of a three-component MEMS accelerometer are different: while the wafer-coplanar are variable gap electrodes, the perpendicular one is a variable area electrode. The latter typology is less sensitive; therefore, to obtain the same sensitivity among all three axes, it is necessary to build this axis over a larger area.

In the following paragraphs, we introduce the theoretical basics of the capacitive MEMS accelerometers, focusing in particular on the analogies between the mechanical and electrical systems and how these are adopted in the realization of the transducers employed in the MEMS sensors. The detailed recall of these topics is also essential to consciously evaluate the present-day capabilities of the MEMS sensors and to depict their possible future developments.

### 2.1. Basics of the Mechanical System

The sensing system of an accelerometer is designed for the detection of inertial forces on the base of a damped spring-mass structure. The system can be described by the equation of motion for a rigid body: the second law of Newton states that the force applied to the body is proportional to the acceleration and the constant of proportionality is the mass:

(1) $$\vec{F}=m\vec{a}$$

Considering a single direction, along the working axis of the device, the vector equation is reduced to a scalar expression:

(2) $$F_{x}=ma_{x}.$$

**Figure 2:**
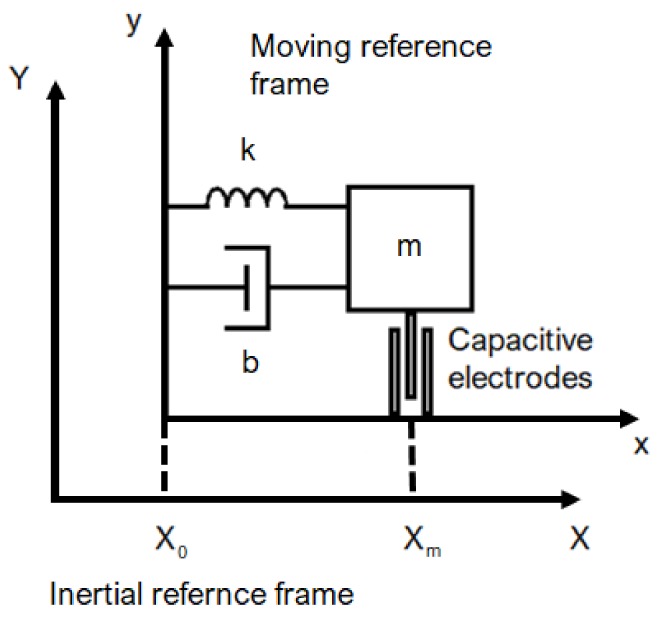
The accelerometer is a spring-mass system attached to a moving reference frame xy; XY is the inertial reference frame. The meaning of *m*, *b*, and *k* is described by Equation (3).

It is possible to use the Newton equations even in a no-inertial system, replacing the reference system. The origin point of the new reference system must be fixed on the device (coordinates of the moving body; Figure 2). It turns that acceleration $a_{x}$, calculated in global coordinates, is replaced with acceleration $\ddot{x}$ calculated in body coordinates.

Equation (2) is then available for the new reference system. The equation of motion for the mass proof in the body coordinate system $x=X_{m}-X_{0}$ is:

(3) $$m\left({\ddot{X}}_{m}-{\ddot{X}}_{0}\right)+b\left({\dot{X}}_{m}-{\dot{X}}_{0}\right)+k\left(X_{m}-X_{0}\right)=-m{\ddot{X}}_{0}.$$

**Figure 3:**
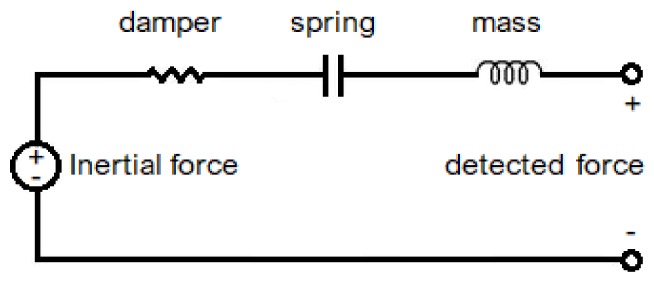
The equivalent circuit of the proof mass accelerometer.

On the left-hand side are the contributions related to the inertial motion of the proof mass, to the damping, and to the linear force of the spring (Figure 2); on the right-hand side is the initial external force, which is often referred to as the apparent, or fictitious, force. The Fourier transform can be used to obtain the mechanical transfer function:

(4) $${\hat{H}}_{j\omega x/a}\left(\omega \right)=\frac{j\omega /\omega_{0}^{2}}{1-{\left(\omega /\omega_{0}\right)}^{2}+2j\zeta \left(\omega /\omega_{0}\right)},$$

where $\omega_{0}=\sqrt{k/m}$ is the mechanical resonant frequency of the suspended proof mass, and $\zeta =b/\sqrt{4km}$ is the damping ratio; the Equation (4) describes the mechanical systems [13]. The analogies with the damping spring mass system can be used to schematize the mechanical system as a resistor capacitor–inductor system; the equivalent circuit diagram is shown in Figure 3.

### 2.2. Modeling of the Capacitive Transducer

The electromechanical transducer is a device characterized by the reversibility property of the electromachine: it is able to transform the mechanical power into the electrical one, and back again. To simplify the explanation of the mathematical model, a lossless system with a single electrode and a single degree of mechanical freedom can be considered and, according to the principle of superposition, the mathematical model of the whole transducer can be obtained later.

**Figure 4:**
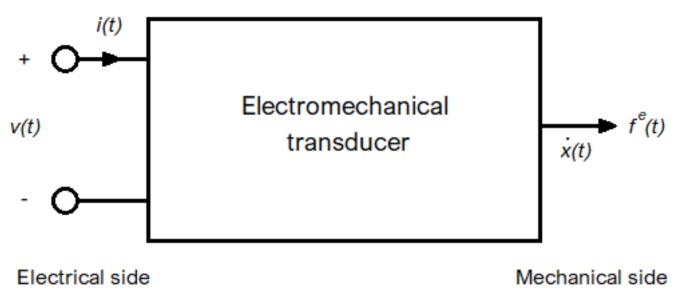
Basic electromechanical transducer with one electrical terminal pair and a single mechanical degree of freedom.

A schematic diagram of a capacitive transducer is shown in Figure 4. On the electrical side, voltage v(t) and current i(t) are time-dependent; on the mechanical side, electrical force $f^{e}\left(t\right)$ and velocity $\dot{x}\left(t\right)$ (where *x* is the position) are time-dependent. By means of these variables, it is possible to obtain both the electric power $p_{e}\left(t\right)=v\left(t\right)i\left(t\right)$ and the mechanical power $p_{m}\left(t\right)=f^{e}\left(t\right)\dot{x}\left(t\right)$. Assuming the absence of any dissipative effects, it gives $p_{e}=p_{m}$. Not all the power is used in the transformation process since part of it is accumulated in the electrostatic form on the capacitor. By assuming a capacitive transducer with an electrically linear response, the capacity C[x(t)] is defined as the linear coefficient between electrical charges q(t) and voltage v(t):

(5) q(t)=C[x(t)]v(t),

where x(t) is the mechanical variable. By means of the principle of charge conservation, the current i(t) and the electric power $p_{e}\left(t\right)$ can be obtained. This is the result of the sum of the electrostatic energy stored in the capacitance $\frac{d}{dt}\left[\frac{C(x)}{2}v^{2}\right]$ and the mechanical power $\frac{1}{2}\frac{dC}{dx}v^{2}\frac{dx}{dt}$. The mechanical power can be also expressed as follows:

(6) $$p_{m}\left(t\right)=\frac{1}{2}\frac{dC}{dx}v^{2}\frac{dx}{dt}=f^{e}\left(t\right)\dot{x}\left(t\right).$$

From Equation (6), the electrostatic force is obtained:

(7) $$f^{e}=\frac{v^{2}}{2}\frac{dC}{dx}.$$

In Equation (7), any variables or mechanical constants appear; this means that the force is solely of electrical origin.

**Figure 5:**
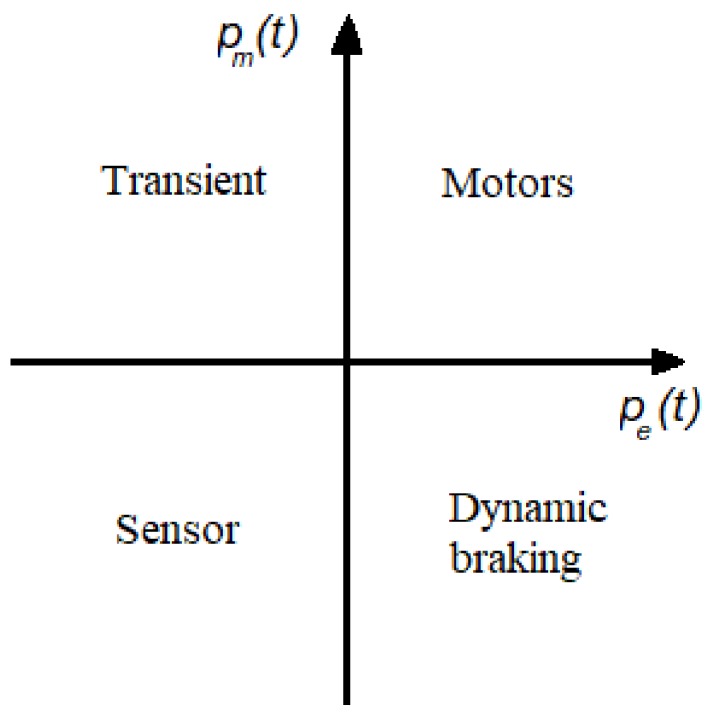
In the plane between electrical power and mechanical power, the capacitive transducers fall in the “sensor” field.

The operating points of these electric machines can be classified according to the four possible combination between temporal average of both electrical and mechanical powers. The capacitive transducers and then any seismic sensor, and also the MEMSs, are characterized by negative $p_{e}\left(t\right)$ and $p_{m}\left(t\right)$: the mechanical power input is converted into electrical power. Therefore, in the plane between electrical and mechanical powers, they fall in the third quadrant (Figure 5).

### 2.3. Capacitive MEMS

The manufacturing of MEMS accelerometers is made using the crystalline Si and a dry reactive ion attachment process. This construction technique was initially borrowed form the ones employed in the realization of the integrated circuits and it is nowadays fairly simple and economical. Only later, some MEMS-specific fabrication processes have been developed. The basic production process is the photolithographic process which involves the “printing” of the pattern of the circuit on the crystalline silicon wafer. Consecutive and repeated steps of substrate carving and etching, together with ion doping to alter the materials’ properties, allow for creating the three-dimensional structure. These processes allow for achieving the complex mechanical pattern of the MEMS geometry which include both fixed and mobile elements. Probably, in the next future, the 3D printing will represent the new approach for the fabrication of miniaturized devices, replacing the other methods, at least for rapid prototyping [14]. For a complete review of the fabrication technologies and materials used for MEMS production, refer to [11].

The mechanical stiffness of the various elements is ensured by a truss structure. The gap between the elements is in the order of few μm and is small if compared to the width of the trusses. The trusses of the mobile elements are electrically conductive, while those of the stationary electrodes are isolated on both sides. The capacitance between a single truss and the Si substrate is very small, so that parasites and contributions of stray are much larger than transducer capacitance. Complex structures that involve higher capacity are preferred because they are less susceptible to parasites.

The sensing part of a MEMS device is interconnected to the circuitry using metal traces that are patterned on the thin dielectric layer. The estimation of the capacitance of the traces is important to counteract their contribution in the parasitic effects and improve the sensitivity. The adjacent electrodes are designed for a given capacitance, but they could interact with the surrounding elements and in particular with substrate. The resulting effect is the lowering of resolution and accuracy.

**Figure 6:**
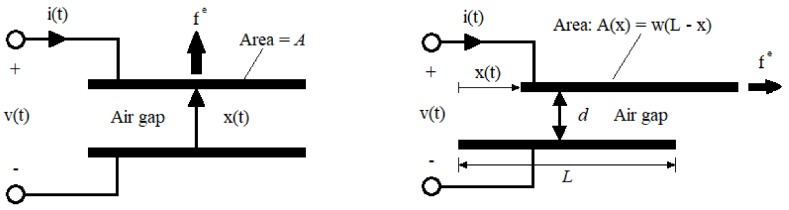
(**Left**): variable-gap capacitor with parallel electrodes of fixed area; if the gap x(t) remains small with respect to all areal dimensions, the fringing fields can be neglected. (**Right**): variable-area capacitor; the air gap is fixed and the area is variable with respect to one degree of freedom. If the gap is small with respect to areal dimensions, fringing can be neglected.

The capacitive MEMS transducers can be classified into two types according to the different designs of their geometry: variable gap and variable area transducers [15] (Figure 6). The first type is characterized by fixed area and variable gap between the capacitors. The electrical constraints imposed on a variable-gap capacitive transducer are the constant charge and the constant voltage. The constant charge constraint gives constant electrical force. The constant voltage constrains the electrical force to have a strong dependence on gap. The second type is characterized by fixed gap and variable area. The constant voltage constraints the electrical force to be constant. The constant charge constrains the electrical force to have a strong dependence on the area. The two types of transducers have different behaviours and they exploit constant charge and constant voltage respectively, to counteract the distribution of the surface charge on the electrodes.

**Figure 7:**
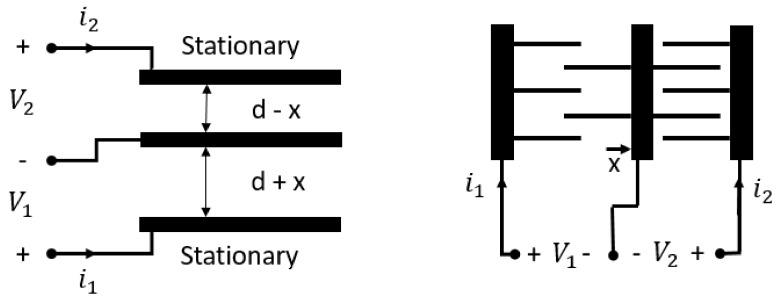
(**Left**): three-plate variable-gap device; (**Right**): three-plate variable-area device.

The comb-drives are electrodes with interdigitated structure which are used in a variety of MEMS actuators. They have high aspect-ratio geometry and are specifically designed to maximize the useable working area. Such geometry is usually adopted for variable area capacitors. In the comb-drive electrodes with single polarity excitation, the moving electrodes undergo a direct force perpendicular to the plane of combs. The main limitation of comb-drive geometry is an unwanted electrostatic force component that pulls the moving electrodes out of alignment. To reduce such parasitic effect, the devices may be operated as three-plates systems in which the middle electrode moves back and forth (Figure 7) using the bipolar voltage excitation [9]. The advantage of the tree-plate systems, operated in a differential mode, is the excellent response linearity between displacement and capacitance charge over a large dynamic range. Moreover, they are immune to the parasitic cable capacitance and most of the three-plates geometries are inherently self shielding, which reduces stray coupling noise. The performance depends on the difference between the two capacitances and improvements in the linear dynamic range of such sensor can be achieved through the cancellation of the second-order terms arising from the capacitance design.

For both the variable-gap and variable-area transducers, the electromechanical force always acts to increase the overall capacitance of the system; this general rule of thumb is always valid and applies to all capacitive electromechanical couplings, irrespective of the geometry. The only way to overcome the tendency of force to increase is to introduce an external constraint such as feedback [15].

The negative feedback is a system designed with the aim to exert a force which opposes the movement of the oscillating mass. The negative feedback affords benefits to the sensor, reducing the nonlinearity of transducers [16]. The force feedback uses the electrostatic force to maintain the moving element at the equilibrium position: when the mass oscillates, it drives the moving element back to the equilibrium. Moreover, the force feedback attenuates the effects imparted by eventual mechanical shocks, when the signal is far larger compared with the normally expected one. To be effective, the feedback response must be sufficiently fast and the feedback voltage must have sufficient voltage range [17,18].

The feedback force balance is realized exactly adopting the comb geometry described above which entails a high aspect ratio; they are usually designed as variable area capacitors. The electric field in the coplanar comb-drive structures is highly non-uniform, meaning that the uniform field approximation cannot be used. The comb-drive and variable-area geometries have electrical force independent of displacement for fixed voltage. The mathematical treatment used to model a capacitive sensor is the coenergy; for a complete review, refer to [19,20].

The electronic circuitry for commercial accelerometers is quite sophisticated. An application-specific integrated circuit (ASIC) is designed to handle not only sensing, but also ratio metric and temperature compensation. It often converts output into various digital formats. Switched capacitance is used to mimic resistors and a modulation scheme suppress noise with constant 1/f. Electronics can be simplified for an analysis with op-amp circuit with feedback capacitance and resistor.

The transfer function relating the current $\hat{i}$ to the output voltage ${\hat{v}}_{0}$ is:

(8) $${\hat{H}}_{v_{0}/i_{0}}\left(\omega \right)=\frac{{\hat{v}}_{0}\left(\omega \right)}{{\hat{i}}_{0}\left(\omega \right)}=\frac{R_{f}}{1+j\omega R_{f}C_{f}}.$$

**Figure 8:**
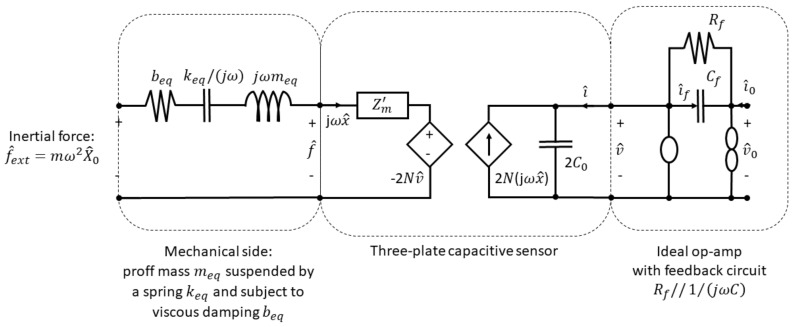
Equivalent circuit of the capacitive MEMS accelerometer.

The transfer function of the sensor is calculated as the product of the subsystems’ transfer functions. The equivalent circuit of the whole system is shown in Figure 8.

The transfer function acceleration *a* to output voltage $v_{0}$ is:

(9) $${\hat{H}}_{v_{0}/a_{0}}\left(\omega \right)\approx -2\frac{1}{\omega_{0}^{2}}V_{o}\frac{dC}{dx}\frac{1}{C_{f}}.$$

In International System (SI) units, the term ${\hat{H}}_{v_{0}/a_{0}}$ in Equation (9) is in V/(m/s${}^{2}$), while in the commercial MEMS accelerometers the sensitivity is usually specified in mV/g where *g* is the gravitational force due to gravity and is *g* = 9.81 m/s${}^{2}$. To convert the units from standard SI units to this most common convention, the term ${\hat{H}}_{v_{0}/a_{0}}\left(\omega \right)$ is multiplied by a factor of $10^{3}/9.81\approx 101.94$.

Since the mechanical transfer function is dependent on air density, the packaging is an integral part of MEMS design. This imposes a design trade-off between the requirements of good sensitivity and a large bandwidth. The mechanical white noise of thermal origin influences the resolution of accelerometers [17,18]. This noise can be represented as an external force contribution related to an integral of the noise density over the bandwidth of the accelerometer.

**Figure 9:**
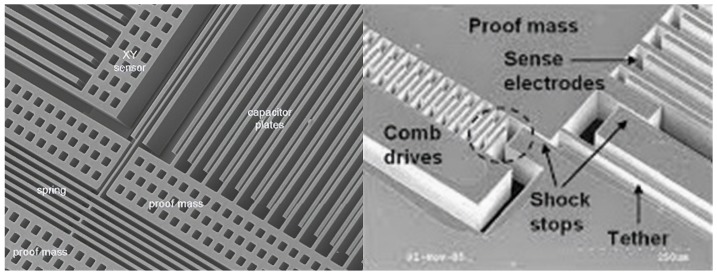
SEM image showing the details of a capacitive MEMS accelerometer.

The selection of the type of electrode (variable-gap or variable-area) imposes the design rules of the MEMS fabrication process. The design rules prescribe a certain minimum value for the gap spacing *d* and a maximum value for *h* (height of the electrode). Assuming the same values both for *d* and *h* in the two design alternatives, the variable-gap configuration is L/d times more sensitive than the variable-area configuration (*L* is the length of the electrode). The typical accelerometer structure features a large number of electrodes and a correspondingly large effective length (Figure 9). Thus, for high-sensitivity (low-g) applications, the variable-gap configuration may be the best choice. Conversely, for low-sensitivity (high-g) applications (e.g., detection of mechanical jolts and shocks), the variable-area structure may be preferred because sensitivity can be traded in favour of a larger bandwidth. The variable-gap configuration is subject to pull-in problems which can be avoided by designing mechanical stops to prevent the distance to be too small if compared to the traction spacing: $d>d_{bs}<d^{*}$ where $d^{*}$ is the traction spacing. The shocks result in small contact areas and the van der Waals forces cannot prevent the spring from restoring the moving electrode around its equilibrium. For this reason, the springs are built with bent beam structures so that the moving test mass is kept in balance.

The sensitivity of the accelerometers is directly proportional to dC/dx and to the bias voltage $V_{B}$; it is inversely proportional to the squared resonant frequency and to the feedback capacitance $C_{f}$. A practical constraint imposed on the MEMS manufactures is that, for any given fabrication process and the associated design rules, the nominal capacitive air gap is fixed. Thus, the only way to increase the derivative dC/dx to increase the net area of the capacitive element, which increases the proof mass. The increasing of the mass decreases the resonant frequency, which, in turn, further increases the sensitivity, to the detriment of the bandwidth.

The study of the stability of capacitive MEMS devices is aimed to the prevention of their failures. It is convenient to look for general criteria to assess the instability of the system. Table 1 summarizes the instability thresholds for both types of transducers. Considering the definition of capacity, it is possible to deduce that only the variable-gap devices can become unstable and only when the voltage is fixed. The instability consists of pull-in phenomena when a threshold value of a voltage between the electrodes is overcome, causing their permanent instability. Pull-in instability can even permanently disable the in variable-gap MEMS devices. Finally, high order effects, noise sources, and distortions may affect the signal. For this reason, it is important to consider the following parameters accordingly to the specific objectives: offset, sensitivity, resolution, nonlinearity, cross-axias sensitivity, temperature sensitivity, ASIC, ratiometricity, and self-test. The treatise of these topics is out of the aim of this review; we therefore refer to the specific literature [13,15,16,19,20]. In this review, we will only report the range of values of some generic parameters desirable for application in seismology.

## 3. Application of Capacitive MEMS Accelerometers to Seismology

Apart from the applications strictly related to earthquake monitoring, MEMS sensors are also employed for closely related topics. The following wide areas of application can be distinguished: (i) earthquake observation and seismological study, (ii) seismic surveys and imaging, and (iii) vibration monitoring of structures and structural assessment. We will introduce the most relevant applications within these groups, and present some cutting-edge applications which have started developing in the most recent years.

The conventional seismic sensors are usually conceived to measure the ground velocity or, alternatively, ground displacement and acceleration These traditional sensors rely on the spring-mass principle in which the inertial oscillations of a damped, bulky mass are converted into an electrical signal. The size of the MEMS devices imposes, first of all, limited sizes and weight; therefore, the proof mass must be extremely reduced. As a consequence, the minimum forcing to induce a detectable electrical output should be greater than a given threshold. For this reason, the MEMS sensors are naturally sensitive to the acceleration in the higher range of the seismic spectrum (strong-motion) and they are commonly referred as “MEMS accelerometers”. Those constructive constraints represent a limit for the development of effective “MEMS velocimeters” which could be better suitable for the “weak-motions”. The available MEMS accelerometers have different technical specifications. The exact requirements depend on the specific application and the following parameter should be considered:

1. the noise output spectral density should have a flat response to the acceleration and be in the order of $10^{-5}$ to $10^{-7}\;$m·s${}^{-2}/\sqrt{\mathrm{Hz}}$;
2. the sensitivity, defined as the ratio between the physical input and the electrical output, should be in the order of $10^{2}$ mV·m${}^{-1}\cdot$s${}^{2}$;
3. the detectable amplitude range should be in the order of $\pm 2\times 10^{0}$ to $\pm 2\times 10^{1}$ m·s${}^{-2}$; however, it ultimately depends on the aims of the application;
4. the bandwidth should overlap, even partially, with the range between $10^{-2}$ and $10^{2}$ Hz;
5. the resolution, defined as the least detectable acceleration, should be in the order of $10^{-2}$−$10^{-3}$ m·s${}^{-2}$.

**Figure 10:**
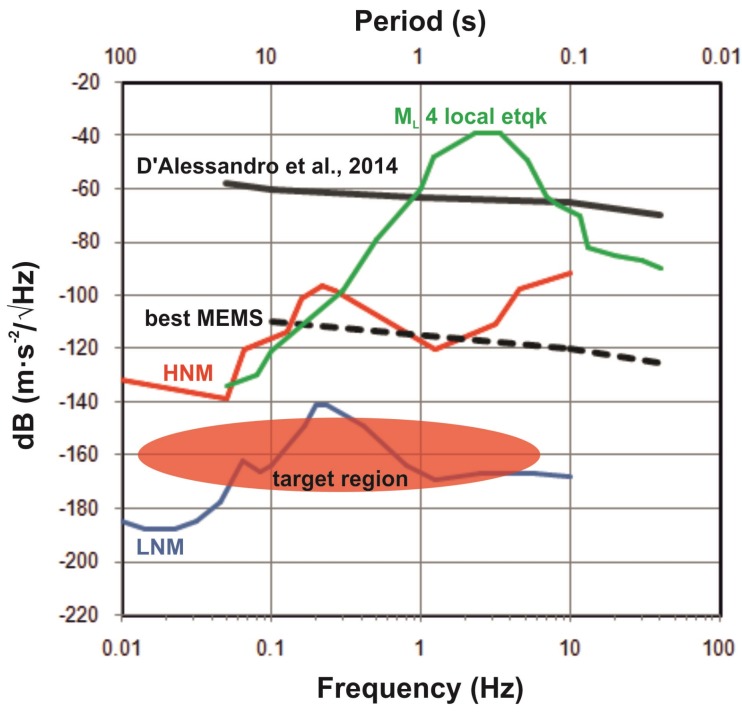
Comparison of the Power Spectral Density (PSD) for some MEMS sensors compared with the seismic noise models (red and blue lines from [37]), and with a spectra response of a local earthquake (green line). The red area indicates the target zone desirable for the next generation of MEMS sensors. Figure from [12].

Figure 10 shows the Power Spectral Density (PSD) of a noise test for the commercial MEMS accelerometer employed for an urban seismic network [21] and for the best MEMS available today. Even the poorer ones are able to detect peaks of acceleration for local earthquakes. The choice of the sensors may be either the result of a selection among the most suitable devices commercially available, or the result of ad hoc design and production. The decision depends on the specific objectives and scale of the purposed application, and on the allocated budget. In those applications encompassing the use of a notable number of sensors, the design of a targeted device could be easily amortized and therefore is even desirable. However, the offer of commercial MEMS is very wide and new products with increased performance are continuously released, making it pretty easy to choose the sensor with the desired requirements for earthquake observation and structural monitoring systems. The effectiveness of several commercial MEMS has been proved by means of various validation techniques [22,23,24,25,26,27,28]. Many studies focused on the accelerometers integrated in the smartphones just because of their large diffusion, even though these are not the best-performing [29,30,31,32,33]. A more accurate characterization should be recommended especially for prototypes; the characteristics should be ideally tested for each single sensor.

Self-noise inevitably affects each sensor and the quality of its measure. The techniques for the characterization of self-noise in the sensors used in seismology, and, in general, in all the inertial sensors are well consolidated and standardized. In particular, the self-noise is the result of the combination of several types of errors. Both the deterministic and the stochastic errors can be analytically or mathematically modeled so that their effects can be mitigated or even entirely eliminated. The assessment of the noise sources, either digital or analog, can be modeled by evaluating the power spectrum (analysis in the frequency domain) or analyzing the time signal (analysis in the time domain) [29,34,35,36]. The causes of the noise (i.e., white noise, random walk, movement noise, quantization noise) can be derived trough the evaluation of the shape of the Power Spectrum Density (PSD) and Allan Variance (AV) curves, known the theoretical link between PSD and AV. In fact, each type of error is characterized by different slope angles of their respective PSD and AV functions in a bi-logarithmic graph [34]. The evaluation and modeling of the errors are essential to compensate and minimize their influence on the performance of the sensor and then increase their reliability.

### 3.1. Earthquake Observation and Seismological Studies

Earthquake observation and related seismological studies are certainly the fields where the resort to MEMS has the most striking impacts. In the last decade, MEMS accelerometers have been more and more exploited for a new generation of seismic monitoring network. The implementation of high-density, real-time, and low-cost networks was also encouraged thanks to the technological development reached in data transmission, computational power, and data storage capability.

The structure of the networks is unchanged with respect to the traditional networks, but owing to their higher noise floor, the MEMS-based seismic stations can also be installed in relatively noisy sites such as urban areas or even inside buildings. Because of this characteristic, such networks are often built on a community participation principle, in which volunteers host in their own place a seismic monitoring station. In their early stages, MEMS-based seismic networks were developed as limited, prototypal networks within universities or research institutions. Over time, some of them developed until they become well-established structures extending both at urban scale and country scale. The networks were mainly established in areas characterized by high seismic risk (e.g., Italy, Japan, Korea, New Zealand, Taiwan, USA), where the combination between seismic hazard and vulnerability is particularly unfavourable (i.e., historical city centres), or where the combination between hazard and exposure is critical (i.e., schools, hospitals, or any other crowded building). Commonly, the ultimate objectives of such conceived networks are: earthquake detection and “now-cast”, intensity mapping for immediate responses for civil protection authorities, and earthquake early warning systems (EEWS).

Here, we describe some of the most relevant cases of earthquake observation networks based on MEMS sensors. Among the first, a network of 25 MEMS accelerometers managed by the Idaho National Engineering and Environmental Laboratory [38], the Quake-Catcher Network (QCN) managed by the University of Stanford [39,40], a project from the Japan Meteorological Agency [41], and the Community Seismic Network (CSN) developed by the California Institute of Technology [42]. In particular, the QCN has spread in several countries and has become a global-scale network [43,44,45,46].

Starting from the 2010s, a multitude of similar projects have been implemented all around the world. Some of them encompass the design, prototyping, and production of devoted MEMS sensors [47,48,49], some focusing on the realization of a complete MEMS-based seismic station exploiting commercial products [50,51,52,53,54,55,56], and some others realize real networks with MEMS-based stations [21,57,58,59,60,61]. Some other projects just exploit the mobile phones themselves by means of specific apps, focusing on the huge diffusion of those devices [31,62,63]. In all the aforementioned cases, the main declared, ultimate task of those networks is the implementation of EEW systems to automatically take timely and targeted actions in case of strong earthquakes [64]. Even though the short period of activity, the MEMS-based seismic networks provided significant results and proved to be reliable enough and especially suitable for both on-site and regional EEW systems. Their capability allows for recording strong (M > 6) regional earthquakes at a distance of few hundreds of km, and even moderate (M∼3) local earthquakes at a distance of the order of some tens of km [21,28].

**Figure 11:**
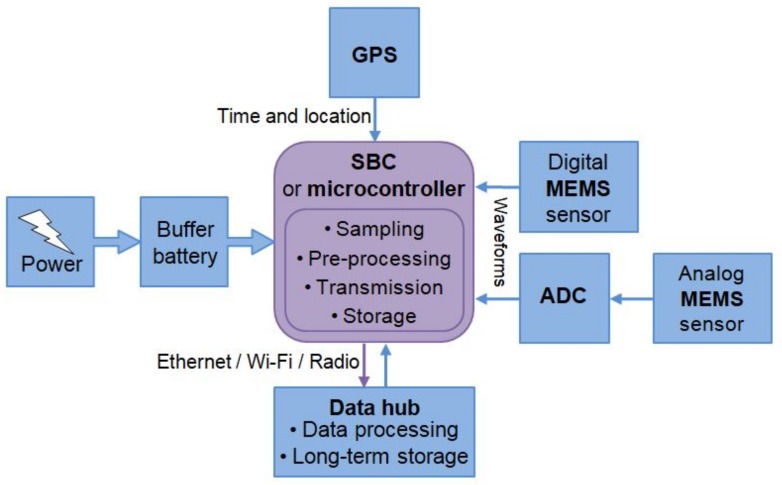
Working scheme of a typical MEMS-based station for seismic monitoring.

The MEMS-based seismic station described in those projects were specifically designed and assembled, and some of them have been commoditized in the very last few years. The criteria to set a MEMS-based seismic station are basically very similar (Figure 11). The core of the system is a microcontroller, or a Single Board Computer (SBC) if more computational power is required (e.g., data processing on site); it manages the main operations. A three-axial MEMS accelerometer is the standard choice; however, several complementary sensors can be integrated in a single station (e.g., angular, magnetic, acoustic, thermal, or pressure sensors) to build a multi-parametric system. Some MEMS have a digital output, some others need the Analog-to-Digital Converter (ADC) to sample the analog signal and provide a digital output to the controlling unit. Considered the frequency range of interest for earthquakes (10${}^{-1}$–$10^{2}$ Hz), the data coming from the MEMS accelerometer must be sampled at the appropriate frequency. In order to reduce aliasing, the sampling rate is recommended between 100 and 200 Hz. A temporal reference is necessary for the synchronization of the signals coming from the various stations and precision in the order of the 10${}^{-3}$ s is desirable. The most reliable way to get a temporal framework is with a GPS; otherwise, the Network Time Protocol (NTP) is also suitable when the internet connection is available. Both the systems widely guarantee the precision required. The transmission of the data are a crucial part: to be really useful for early damage assessment, and even more for early warnings, the stations must transfer information in real time. The transmission systems can be various; a differentiation of the typologies of transmission is desirable in order to guarantee the continuity in case one of them fails. Then, data must be collected and managed into a main hub (i.e., seismic room). Finally, the station needs low voltage DC power supply and the consumption is very low. An opportune buffer battery can guarantee in case of temporal power interruption.

The use of MEMS-based networks for more specific seismological applications (i.e., localization, magnitude estimation, etc.) is conversely not really robust at present, mainly because the data accuracy requested for such tasks is certainly higher. In fact, rougher information about the arrivals or about the amplitude of seismic waves would result in relevant uncertainties in the estimation of the location of a given earthquake or into a weak assessment of its energy release (i.e., earthquake magnitude) although some results are encouraging [65,66,67]. However, MEMS stations could contribute for temporary network tightening in case of seismic crisis when a fast monitoring enhancement around the epicentral area is desirable, also with the help of local citizens [43,44,68]. Moreover, in the cases when a certain degree of redundancy is required (e.g., diversification of the instruments or of the transmission protocols), then a mixed traditional-MEMS network could represent a proper solution in terms of cost–benefit ratio [28,67,69,70,71].

### 3.2. Seismic Surveys and Imaging

Another field of application of MEMS accelerometers is the seismic surveying and imaging both for deep (i.e., oil and gas exploration) and shallow (e.g., near surface geophysics) investigation; fundamentally, they are suitable for the active techniques, i.e., those involving an artificial source for generating the seismic waves. With respect to the traditional, the MEMS sensors might be preferable because of their reduced dimension and weight, being easier to handle, and also better in the long-term endurance [72]. In this field, either the commercial products, or specifically designed devices can be used. Moreover, the use of MEMSs can indirectly improve the quality of the seismic imaging: a huge array of sensors (hundreds to thousands) can be deployed at the same time with contained cost and acceptable quality, resulting in high-resolution geophysical models.

Since the mid-2000s, numerous papers compare the traditional, one component coil geophones employed in the geophysical exploration with several types of MEMS accelerometers or examined the results after lab and field experimental tests [73,74,75,76,77,78,79,80,81,82,83,84,85,86]. For the early generations of sensors, the results were not so favourable; later, the results indicate, all things considered, better performance for active seismic surveys. Benefits mainly involve the passage from analog 1C sensors, to digital (non-requiring digitizer) 3C ones. The amplitude response is more accurate over a broader bandwidth. Considering the spectrum of the seismic noise, MEMS accelerometers give better results at the higher frequency, while the traditional geophones are still better in the lower frequency range (below 1 Hz).

### 3.3. Vibration Monitoring and Damage Assessment of Structures

The Structural Health Monitoring (SHM) is a fundamental tool to integrate and support conservation strategies of infrastructures and to preserve their strategic function (i.e., security, management, organization). Buildings, and any infrastructure in general, are built to stand for ordinary and extreme events. The stress factors acting on the structures can be due to natural or anthropogenic factors: seismic events, atmospheric agents (wind, thermal cycles), vibration due to traffic flow, and applied loads. They all contribute to lower the resistance properties (corrosion, alteration, etc.).

The effects of such factors are today examined by means of various types of sensors [87,88]. At present, monitoring is often carried out for short periods because of the costs and of the logistic difficulties to maintain long-term campaigns; this inevitably leads to poor technical and scientific results. It is about making a choice between technical-scientific needs and the financial affordability and, again, MEMS technology helps to overcome such a compromise. Not only the cost of the sensors can be drastically cut, but also the cost to wire a building because the miniaturized MEMS-based monitoring stations can be easily connected wireless and manage real-time data [89,90,91].

Several recent projects encompass the realization of prototypes of MEMS-based (mainly accelerometers and gyroscopes) monitoring station specifically designed for SHM: they are based on the measure of the structural vibration, from which structural health and post-event (e.g., earthquake) damage can be diagnosed [22,23,61,92,93,94,95,96,97,98,99]. Other studies also investigated the possibility to use the MEMS accelerometers integrated within the smartphones to develop citizen-engaging networks for SHM, like the ones created for earthquake observation and EEW [30,62,100,101].

Validations are performed with scaled experiments on lab shaking tables, full scale test on shaking platforms, or empirical evaluations in comparison with traditional sensors. The main target buildings are structures relevant for their historical and cultural value or crucial infrastructure for safety or transports. All these systems enable the remote monitoring of constructions; the compilation of a register of historical data, the creation of files for post-processing; and establish computer-based protocols for evaluation of information, defining automatic alarms when the monitored data exceed given thresholds. A reliable modeling of the structure behavior is still an open research issue and the extensive and systematic monitoring of buildings possible with MEMS sensors can also provide relevant data for the support and the implementation of robust modal analysis or finite element analytical models [97].

### 3.4. Other Applications

An emerging application of MEMS sensors is rotational seismology. In addition to the translational motion, earthquakes generate also a rotational one, especially in the near-field. The simultaneous measurement of all the six components of motion is essential to characterize thoroughly the ground shaking and also to refine the mechanical models of the seismic rupture which account only the translational components [102]. Even more essential is the evaluation of the rotational components for buildings and structures. The considerable cost of rotational sensors represented the main limitation for the development of dedicated networks. Rather, MEMS accelerometers and gyroscopes specifically designed could represent a solution with an acceptable price/performance ratio.

The first observation of the rotational components using a commercial MEMS sensor is presented in [103]. More recently, some other studies proposed prototypes of angular MEMS sensors. In particular, [104,105] designed and characterized the performance of an electrochemical sensors based on the molecular-electronic transfer (MET) technology, today considered as one of the most promising technologies for the measurement of angular velocity. Others [106] realized a capacitive MEMS rotational sensor, while [107] designed a capacitive sensor that combine both angular and linear (acceleration) sensing.

Another side application of MEMS technology regards measurements of the gravity acceleration. Gravity anomalies are related to irregular distribution of rock density and therefore could be really useful for oil and gas exploration, detection of buried fault, and in general of any crustal discontinuity. The measure of the gravitational acceleration requires devices able to guarantee high sensitivity and high stability over time. For these reasons, gravimeters are extremely bulky and expensive instruments. MEMS accelerometer can only operate as a seismometer because they cannot reach the required sensitivity at very low frequency, but recent technical development made possible some steps toward MEMS gravity sensors. The use of an anti-spring system, in which the spring become softer with increasing displacement, allowed the lowering of the resonant frequency of the sensor. In this way, it is even possible to measure signals with frequency in the order of 10${}^{-5}$ Hz. Such conceived sensors have been able to detect the Earth tides [108,109], and experimental field tests confirmed the possibility to used them as portable devices for gravity imaging [110,111].

## 4. Future Perspectives

After the extraordinary development of MEMS applied to all kind of technological fields, during the mid-2000s, the limits of the technical development of MEMS accelerometers have been considered as almost reached [112]. Although the physical limits exist and are insurmountable, in the very last years, the research in this field has always been able to find new solutions to cope with them. Such solutions involve the optimization of existing technologies, the development of new materials, or the implementation of innovative production processes. Moreover, indications in the development of the future sensors can also be suggested by a rigorous assessment of the failures that affected MEMS sensors [113].

Data for pure seismological studies require the highest possible fidelity, but, currently, MEMS sensors fail to provide data with the requested reliability. However, for more flexible seismology-related applications, they already today represent a good option and will probably be the best one in the future. The capability of the next generations of sensors will allow also for assessing the seismic background noise, detecting signals with acceleration density in the order of a few tenths of ng$/\sqrt{\mathrm{Hz}}$ (target zone in Figure 10). Probably the main success for the very next generation of MEMS sensor for seismology would be to reach high sensitivity into the lower part of the earthquake frequency spectrum (∼0.01 Hz) [114]. The contributions in this direction are numerous in the last several years, even though improvements and optimization are possible for capacitive-based sensors [115,116,117,118]. It will probably be necessary to abandon these types of sensors in the place of devices based on other physical principles [2]. High-precision (nano-g) optical MEMS accelerometers were proposed [119], but likely the most promising technology will be a new generation of electrochemical sensors [1,120,121,122,123,124,125,126,127,128], optical [108,129], or resonant accelerometers [99,130]. In some cases, the proposed solutions imply to the detriment of dimension (from ca. few μm to ca. hundreds of μm) in order to increase the involved physical quantities; however, they still remain micro-sensors.

The potential outcomes coming from the bandwidth extension are huge, with immediate consequences in all the applications fields listed in the previous paragraphs. MEMS are considered the future innovation for country-scale networks in a 10-year outlook [131], focused not only to map the intensity distribution or to early warning systems, but also to proper seismological studies. Similarly, the great diffusion of smartphone equipped with MEMS and devoted apps will enable the development of human-centred, global-scale, seismic networks involving the participation of the citizens [132,133,134].

## 5. Conclusions

In this review, we provided the state-of-the-art of the role of capacitive MEMS in seismology and related disciplines. MEMS are small, low power, durable, and, above all, cheap devices enabling a wide range of applications in terms of scale and variety of recorded signals. MEMS sensors were revealed to be very important for the most recent developments in seismology-related disciplines. They enabled the implementation of several systems that otherwise would have been just impossible to realize, mainly because of the huge costs. In fact, high density seismic networks or high detail structural monitoring were only rarely encountered in the past. In the most recent years, MEMS-based applications have been greatly emerging, especially those devoted to earthquake early warning systems and earthquake intensity mapping.

Despite the progress reached in the last decade, the performance of MEMS sensors is still not comparable to the traditional devices. The self-noise of the MEMS accelerometer will be likely reduced in the next generations of sensor, so that also part of the seismic background noise could be assessed (Figure 10). Another disadvantage is the relatively poor response at low frequencies; this is the reason that MEMS sensors are especially suitable for strong-motion seismology these days. However, the most recent developments are promising and, likely, in the very next future the sensors offering good performances at lower frequency will broaden the range of application. The improvements direction is towards smart buildings and smart cities, where the health of all the structures is continuously monitored and the information shared in real time to support local-and-regional-scale EEW systems. In addition, side-applications like rotational seismology, seismic imaging, and gravity measurement will grow more and more.

The growing phase of such technology is just started. It will also involve the availability of a huge amount of data representing an unprecedented opportunity for scientists and technicians to make progress in seismological studies, earthquake engineering, and geotechnical engineering.

## Figures and Tables

**Table 1:** The stability of basic variable-gap and variable-area capacitive transducers.

Electrical Constraint	Basic Variable-Gap Capacitor: $\frac{d^{2}C}{{\mathrm{dx}}^{2}}>0$	Basic Variable-Area Capacitor: $\frac{d^{2}C}{{\mathrm{dx}}^{2}}=0$
Constant charge: i=0	Always stable; no effects on system stiffness	Always stable; electromechanical coupling stiffness and increased resonant frequency
Constant voltage: v=0	Electromechanical coupling decreases the resonant frequency; pull-in instability if $|v_{0}|>\sqrt{2k/d^{2}C/dx^{2}}$	Always stable; no effects on system stiffness

## References

[1] Havskov J., Alguacil G. (2004). Instrumentation in Earthquake Seismology.

[2] Havskov J., Alguacil G. (2016). Seismic sensors. Instrumentation in Earthquake Seismology.

[3] Roylance L.M., Angell J.B. (1979). A batch-fabricated silicon accelerometer. IEEE Trans. Electron Devices.

[4] Barlian A.A., Howe R.T., Kovacs G.T., Pruitt B.L. Micro and Nanoscale Education at Stanford University. Proceedings of the Solid-State Sensors, Actuators, and Microsystems Workshop.

[5] Eddy D.S., Sparks D.R. (1998). Application of MEMS technology in automotive sensors and actuators. Proc. IEEE.

[6] Maluf N., Williams K. (2004). Introduction to Microelectromechanical Systems Engineering.

[7] Tsuchiya T. Technologies, applications, and reliabilities of microelectromechanical systems (MEMS). Proceedings of the 9th SEGJ International Symposium.

[8] Mishra M.K., Dubey V., Mishra P., Khan I. (2019). MEMS Technology: A Review. J. Eng. Res. Rep..

[9] Tang W.C. MEMS applications in space exploration. Proceedings of the Micromachined Devices and Components III.

[10] Kukkonen C.A., Venneri S. (1997). Microsensors and microinstruments for space science and exploration. Space Technol..

[11] Judy J.W. (2001). Microelectromechanical systems (MEMS): Fabrication, design and applications. Smart Mater. Struct..

[12] Scudero S., D’Alessandro A., Greco L., Vitale G. MEMS technology in seismology: A short review. Proceedings of the 2018 IEEE International Conference on Environmental Engineering (EE).

[13] Yazdi N., Ayazi F., Najafi K. (1998). Micromachined inertial sensors. Proc. IEEE.

[14] Li F., Macdonald N.P., Guijt R.M., Breadmore M.C. (2019). Increasing the functionalities of 3D printed microchemical devices by single material, multimaterial, and print-pause-print 3D printing. Lab Chip.

[15] Jones T.B., Nenadic N.G. (2013). Electromechanics and MEMS.

[16] Sedra A.S., Smith K.C. (1998). Microelectronic Circuits.

[17] Lemkin M.A., Boser B.E., Auslander D., Smith J.H. A 3-axis force balanced accelerometer using a single proof-mass. Proceedings of the International Solid State Sensors and Actuators Conference (Transducers’ 97).

[18] Jono K., Minami K., Esashi M. (1995). An electrostatic servo-type three-axis silicon accelerometer. Meas. Sci. Technol..

[19] Melcher J.R., Woodson H.H. (1968). Electromechanical dynamics. Part I: Discrete Systems.

[20] Tilmans H.A. (1996). Equivalent circuit representation of electromechanical transducers: I. Lumped-parameter systems. J. Micromech. Microeng..

[21] D’Alessandro A., Luzio D., D’Anna G. (2014). Urban MEMS based seismic network for post-earthquakes rapid disaster assessment. Adv. Geosci..

[22] Trapani D., Biasi N., De Cecco M., Zonta D. Validation of MEMS acceleration measurements for seismic monitoring with LVDT and vision system. Proceedings of the 2012 IEEE Workshop on Environmental Energy and Structural Monitoring Systems (EESMS).

[23] Trapani D., Zonta D., Molinari M., Amditis A., Bimpas M., Bertsch N., Spiering V., Santana J., Sterken T., Torfs T. Full-scale laboratory validation of a MEMS-based technology for post-earthquake damage assessment. Proceedings of the 15th World Conference on Earthquake Engineering (15 WCEE).

[24] Zou X., Thiruvenkatanathan P., Seshia A.A. (2014). A seismic-grade resonant MEMS accelerometer. J. Microelectromech. Syst..

[25] Acar C., Shkel A.M. (2003). Experimental evaluation and comparative analysis of commercial variable-capacitance MEMS accelerometers. J. Micromech. Microeng..

[26] Evans J., Allen R.M., Chung A., Cochran E., Guy R., Hellweg M., Lawrence J. (2014). Performance of several low-cost accelerometers. Seismol. Res. Lett..

[27] Fougerat A., Guérineau L., Tellier N. (2018). High-quality signal recording down to 0.001 Hz with standard MEMS accelerometers. SEG Technical Program Expanded Abstracts 2018.

[28] Nof R.N., Chung A.I., Rademacher H., Dengler L., Allen R.M. (2019). MEMS Accelerometer Mini-Array (MAMA): A Low-Cost Implementation for Earthquake Early Warning Enhancement. Earthq. Spectra.

[29] D’Alessandro A., Vitale G., Scudero S., D’Anna R., Costanza A., Fagiolini A., Greco L. Characterization of MEMS accelerometer self-noise by means of PSD and Allan Variance analysis. Proceedings of the 2017 7th IEEE International workshop on advances in sensors and interfaces (IWASI).

[30] Feng M., Fukuda Y., Mizuta M., Ozer E. (2015). Citizen sensors for SHM: Use of accelerometer data from smartphones. Sensors.

[31] Kong Q., Allen R.M., Schreier L., Kwon Y.W. (2016). MyShake: A smartphone seismic network for earthquake early warning and beyond. Sci. Adv..

[32] Kos A., Tomažič S., Umek A. (2016). Evaluation of smartphone inertial sensor performance for cross-platform mobile applications. Sensors.

[33] D’Alessandro A., D’Anna G. (2013). Suitability of low-cost three-axis MEMS accelerometers in strong-motion seismology: Tests on the LIS331DLH (iPhone) accelerometer. Bull. Seismol. Soc. Am..

[34] Board I. (1998). IEEE Standard Specification Format Guide and Test Procedure for Single-Axis Interferometric Fiber Optic Gyros.

[35] Vukmirica V., Trajkovski I., Asanovic N. (2018). Two methods for the determination of inertial sensor parameters. Methods.

[36] Quinchia A., Falco G., Falletti E., Dovis F., Ferrer C. (2013). A comparison between different error modeling of MEMS applied to GPS/INS integrated systems. Sensors.

[37] Peterson J. (1993). Observations and Modeling of Background Seismic Noise.

[38] Holland A. (2003). Earthquake data recorded by the MEMS accelerometer: Field testing in Idaho. Seismol. Res. Lett..

[39] Cochran E., Lawrence J., Christensen C., Chung A. (2009). A novel strong-motion seismic network for community participation in earthquake monitoring. IEEE Instrum. Meas. Mag..

[40] Cochran E.S., Lawrence J.F., Christensen C., Jakka R.S. (2009). The quake-catcher network: Citizen science expanding seismic horizons. Seismol. Res. Lett..

[41] Horiuchi S., Horiuchi Y., Yamamoto S., Nakamura H., Wu C., Rydelek P.A., Kachi M. (2009). Home seismometer for earthquake early warning. Geophys. Res. Lett..

[42] Clayton R.W., Heaton T., Chandy M., Krause A., Kohler M., Bunn J., Guy R., Olson M., Faulkner M., Cheng M. (2012). Community seismic network. Ann. Geophys..

[43] Chung A., Neighbors C., Belmonte A., Miller M., Sepulveda H.H., Christensen C., Jakka R., Cochran E., Lawrence J. (2011). The Quake-Catcher Network rapid aftershock mobilization program following the 2010 M 8.8 Maule, Chile earthquake. Seismol. Res. Lett..

[44] Chung A.I., Cochran E.S., Kaiser A.E., Christensen C.M., Yildirim B., Lawrence J.F. (2015). Improved rapid magnitude estimation for a community-based, low-cost MEMS accelerometer network. Bull. Seismol. Soc. Am..

[45] Yildirim B., Cochran E.S., Chung A., Christensen C.M., Lawrence J.F. (2015). On the Reliability of Quake-Catcher Network Earthquake Detections. Seismol. Res. Lett..

[46] Lawrence J.F., Cochran E.S., Chung A., Kaiser A., Christensen C.M., Allen R., Baker J.W., Fry B., Heaton T., Kilb D. (2014). Rapid earthquake characterization using MEMS accelerometers and volunteer hosts following the M 7.2 Darfield, New Zealand, earthquake. Bull. Seismol. Soc. Am..

[47] Zheng H., Shi G., Zeng T., Li B. Wireless earthquake alarm design based on MEMS accelerometer. Proceedings of the 2011 International Conference on Consumer Electronics, Communications and Networks (CECNet).

[48] Wu J., Liang J., Szu H. 3D MEMS sensor for application on earthquakes early detection and Nowcast. Proceedings of the Sensing and Analysis Technologies for Biomedical and Cognitive Applications 2016.

[49] Wu W., Li Z., Liu J., Fan J., Tu L. A nano-g MEMS accelerometer for earthquake monitoring. Proceedings of the 2017 19th International Conference on Solid-State Sensors, Actuators and Microsystems (TRANSDUCERS).

[50] Rohmanuddin M., Budi E.M., Ferdiana F. Development of seismic sensor application using micro electromechanical systems. Proceedings of the 2011 2nd International Conference on Instrumentation Control and Automation.

[51] Hoque R., Hassan S., Sadaf M.A., Galib A., Karim T.F. Earthquake monitoring and warning system. Proceedings of the 2015 International Conference on Advances in Electrical Engineering (ICAEE).

[52] Zhiqun L., Huang S., Jianru W. (2014). Development on Seismic Sensor System with MEMS Technology for Elevator’s Seismic Condition. Appl. Mech. Mater..

[53] Peng C., Chen Y., Chen Q., Yang J., Wang H., Zhu X., Xu Z., Zheng Y. (2017). A new type of tri-axial accelerometers with high dynamic range MEMS for earthquake early warning. Comput. Geosci..

[54] Pierleoni P., Marzorati S., Ladina C., Raggiunto S., Belli A., Palma L., Cattaneo M., Valenti S. (2018). Performance Evaluation of a Low-Cost Sensing Unit for Seismic Applications: Field Testing During Seismic Events of 2016–2017 in Central Italy. IEEE Sens. J..

[55] Wargantiwar N., Barbati A., Shingade A., Shire A. (2017). Wireless earthquake alarm design based on MEMS accelerometer. Int. Adv. Res. J. Sci. Eng. Technol..

[56] Fu J., Li Z., Meng H., Wang J., Shan X. (2019). Performance Evaluation of Low-Cost Seismic Sensors for Dense Earthquake Early Warning: 2018–2019 Field Testing in Southwest China. Sensors.

[57] Wu Y.M., Chen D.Y., Lin T.L., Hsieh C.Y., Chin T.L., Chang W.Y., Li W.S., Ker S.H. (2013). A high-density seismic network for earthquake early warning in Taiwan based on low cost sensors. Seismol. Res. Lett..

[58] Wu Y.M. (2015). Progress on development of an earthquake early warning system using low-cost sensors. Pure Appl. Geophys..

[59] Kim Y., Kang T.S., Rhie J. (2017). Development and Application of a Real-Time Warning System Based on a MEMS Seismic Network and Response Procedure for the Day of the National College Entrance Examination in South Korea. Seismol. Res. Lett..

[60] D’Alessandro A., D’Anna R., Greco L., Passafiume G., Scudero S., Speciale S., Vitale G. Monitoring Earthquake through MEMS Sensors (MEMS project) in the town of Acireale (Italy). Proceedings of the 2018 IEEE International Symposium on Inertial Sensors and Systems (INERTIAL).

[61] D’Alessandro A., Vitale G., Scudero S., D’Anna R., Passafiume G., Greco L., Speciale S., Patanè D., Torrisi O., Di Prima S. Real-time urban seismic network and structural monitoring by means of accelerometric sensors: Application to the historic buildings of Catania (Italy). Proceedings of the 2018 IEEE International Conference on Environmental Engineering (EE).

[62] Kong Q., Allen R.M., Kohler M.D., Heaton T.H., Bunn J. (2018). Structural health monitoring of buildings using smartphone sensors. Seismol. Res. Lett..

[63] Finazzi F. (2016). The earthquake network project: Toward a crowdsourced smartphone-based earthquake early warning system. Bull. Seismol. Soc. Am..

[64] Satriano C., Wu Y.M., Zollo A., Kanamori H. (2011). Earthquake early warning: Concepts, methods and physical grounds. Soil Dyn. Earthq. Eng..

[65] Saunders J.K., Goldberg D.E., Haase J.S., Bock Y., Offield D.G., Melgar D., Restrepo J., Fleischman R.B., Nema A., Geng J. (2016). Seismogeodesy using GPS and low-cost MEMS accelerometers: Perspectives for earthquake early warning and rapid response. Bull. Seismol. Soc. Am..

[66] Ding W., Liao C., Wang H. (2017). MEMS-based seismic intensity instrument for earthquake early warning. Int. J. Comput. Sci. Eng..

[67] Jan J., Chao W.A., Wu Y.M., Chen C.C., Lin C.H. (2017). How Well Can We Extract the Permanent Displacement from Low-Cost MEMS Accelerometers?. Sensors.

[68] Boaga J., Casarin F., De Marchi G., Valluzzi M.R., Cassiani G. (2018). 2016 Central Italy Earthquakes Recorded by Low-Cost MEMS-Distributed Arrays. Seismol. Res. Lett..

[69] Clayton R.W., Heaton T., Kohler M., Chandy M., Guy R., Bunn J. (2015). Community seismic network: A dense array to sense earthquake strong motion. Seismol. Res. Lett..

[70] Tanircan G., Alcik H., Beyen K. (2018). Reliability of MEMS accelerometers for instrumental intensity mapping of earthquakes. Ann. Geophys..

[71] Anthony R.E., Ringler A.T., Wilson D.C., Wolin E. (2018). Do low-cost seismographs perform well enough for your network? An overview of laboratory tests and field observations of the OSOP Raspberry Shake 4D. Seismol. Res. Lett..

[72] Farine M., Thorburn N., Mougenot D. (2004). General application of MEMS sensors for land seismic acquisition—Is it time. Lead. Edge.

[73] Ronen S., Comeaux L., Cartwright M., Gibson J., Burnett R., Roy J., Watt H. (2005). Comparison between geophones and two MEMS types and repeatability of land data. SEG Technical Program Expanded Abstracts 2005.

[74] Lawton D.C., Bertram M.B., Margrave G.F., Gallant E.V. (2006). Comparisons between data recorded by several 3-component coil geophones and a MEMS sensor at the Violet Grove monitor seismic survey. CREWES Res. Rep..

[75] Laine J., Mougenot D. Benefits of MEMS based seismic accelerometers for oil exploration. Proceedings of the TRANSDUCERS 2007–2007 International Solid-State Sensors, Actuators and Microsystems Conference.

[76] Laine J., Mougenot D. (2014). A high-sensitivity MEMS-based accelerometer. Lead. Edge.

[77] Hauer G., Hons M., Stewart R., Lawton D., Bertram M. (2008). Field data comparison: 3C-2D data acquisition with geophones and accelerometers. SEG Technical Program Expanded Abstracts 2008.

[78] Hons M., Stewart R., Lawton D., Bertram M., Hauer G. (2008). Field data comparisons of MEMS accelerometers and analog geophones. Lead. Edge.

[79] Stotter C., Angerer E., Herndler E. (2008). Comparison of single sensor 3C MEMS and conventional geophone arrays for deep target exploration. SEG Technical Program Expanded Abstracts 2008.

[80] Aizawa T., Kimura T., Matsuoka T., Takeda T., Asano Y. (2009). Application of MEMS accelerometer to geophysics. Int. J. JCRM.

[81] Andò B., Baglio S., L’Episcopo G., Marletta V., Savalli N., Trigona C. (2011). A BE-SOI MEMS for inertial measurement in geophysical applications. IEEE Trans. Instrum. Meas..

[82] Milligan D.J., Homeijer B.D., Walmsley R.G. An ultra-low noise MEMS accelerometer for seismic imaging. Proceedings of the IEEE Sensors.

[83] Zhang Z., Wu J., Bernard S., Walmsley R.G. Chip on Board development for a novel MEMS accelerometer for seismic imaging. Proceedings of the 2012 IEEE 62nd Electronic Components and Technology Conference.

[84] Wei J. (2013). Comparing the MEMS accelerometer and the analog geophone. Lead. Edge.

[85] Brodic B., Malehmir A., Juhlin C., Dynesius L., Bastani M., Palm H. (2015). Multicomponent broadband digital-based seismic landstreamer for near-surface applications. J. Appl. Geophys..

[86] Pike W., Calcutt S., Standley I., Mukherjee A., Temple J., Warren T., Charalambous C., Liu H., Stott A., McClean J. A silicon seismic package (SSP) for planetary geophysics. Proceedings of the 47th Lunar and Planetary Science Conference.

[87] Moreno-Gomez A., Perez-Ramirez C.A., Dominguez-Gonzalez A., Valtierra-Rodriguez M., Chavez-Alegria O., Amezquita-Sanchez J.P. (2017). Sensors used in structural health monitoring. Archives of Computational Methods in Engineering.

[88] Sony S., Laventure S., Sadhu A. (2019). A literature review of next-generation smart sensing technology in structural health monitoring. Struct. Control Health Monit..

[89] Noel A.B., Abdaoui A., Elfouly T., Ahmed M.H., Badawy A., Shehata M.S. (2017). Structural health monitoring using wireless sensor networks: A comprehensive survey. IEEE Commun. Surv. Tutor..

[90] Sabato A., Niezrecki C., Fortino G. (2017). Wireless MEMS-based accelerometer sensor boards for structural vibration monitoring: A review. IEEE Sens. J..

[91] Ragam P., Sahebraoji N.D. (2019). Application of MEMS-based accelerometer wireless sensor systems for monitoring of blast-induced ground vibration and structural health: A review. IET Wirel. Sens. Syst..

[92] Pozzi M., Zonta D., Trapani D., Athanasopoulos N., Amditis A., Bimpas M., Garetsos A., Stratakos Y., Ulieru D. (2011). MEMS-based sensors for post-earthquake damage assessment. Journal of Physics: Conference Series.

[93] Liang Q., Tani A., Yamabe Y. (2015). Fundamental Tests on a Structural Health Monitoring System for Building Structures Using a Single-board Microcontroller. J. Asian Archit. Build. Eng..

[94] Potenza F., Federici F., Lepidi M., Gattulli V., Graziosi F., Colarieti A. (2015). Long-term structural monitoring of the damaged Basilica S. Maria di Collemaggio through a low-cost wireless sensor network. J. Civ. Struct. Health Monit..

[95] Yin R.C., Wu Y.M., Hsu T.Y. Application of the low-cost MEMS-type seismometer for structural health monitoring: A pre-study. Proceedings of the 2016 IEEE International Instrumentation and Measurement Technology Conference Proceedings.

[96] Andò B., Baglio S., Pistorio A. (2018). A low cost multi-sensor system for investigating the structural response of buildings. Ann. Geophys..

[97] Bedon C., Bergamo E., Izzi M., Noè S. (2018). Prototyping and validation of MEMS accelerometers for structural health monitoring—The case study of the Pietratagliata cable-stayed bridge. J. Sens. Actuator Netw..

[98] Fu Y., Hoang T., Mechitov K., Kim J., Zhang D., Spencer B. (2018). Sudden Event Monitoring of Civil Infrastructure Using Demand-Based Wireless Smart Sensors. Sensors.

[99] Hsu T.Y., Yin R.C., Wu Y.M. (2018). Evaluating post-earthquake building safety using economical MEMS seismometers. Sensors.

[100] Castellanos-Toro S., Marmolejo M., Marulanda J., Cruz A., Thomson P. (2018). Frequencies and damping ratios of bridges through Operational Modal Analysis using smartphones. Constr. Build. Mater..

[101] Ozer E., Feng M., Feng D. (2015). Citizen sensors for SHM: Towards a crowdsourcing platform. Sensors.

[102] Lee W.H., Igel H., Trifunac M.D. (2009). Recent advances in rotational seismology. Seismol. Res. Lett..

[103] Nigbor R.L. (1994). Six-degree-of-freedom ground-motion measurement. Bull. Seismol. Soc. Am..

[104] Egorov E.V., Egorov I.V., Agafonov V.M. (2015). Self-noise of the MET angular motion seismic sensors. J. Sensors.

[105] Agafonov V.M., Neeshpapa A.V., Shabalina A.S. (2014). Electrochemical seismometers of linear and angular motion. Encycl. Earthq. Eng..

[106] Projetti M., Vancauwenberghe O., Paulson H., Goujon N., Marty F., Aubry D. Development of a MEMS rotation sensor for oilfield applications. Proceedings of the IEEE Sensors.

[107] Liu H., Pike W. (2016). A micromachined angular-acceleration sensor for geophysical applications. Appl. Phys. Lett..

[108] Middlemiss R., Samarelli A., Paul D., Hough J., Rowan S., Hammond G. (2016). Measurement of the Earth tides with a MEMS gravimeter. Nature.

[109] Prasad A., Bramsiepc S., Middlemiss R., Hough J., Rowan S., Hammond G., Paul D. A Portable MEMS Gravimeter for the Detection of the Earth Tides. Proceedings of the 2018 IEEE SENSORS.

[110] Middlemiss R.P., Bramsiepe S.G., Douglas R., Hild S., Hough J., Paul D.J., Samarelli A., Rowan S., Hammond G.D. (2018). Microelectromechanical system gravimeters as a new tool for gravity imaging. Philos. Trans. R. Soc. A Math. Phys. Eng. Sci..

[111] Hammond G. MEMS gravity sensors for imaging density anomalies. Proceedings of the Optical Trapping and Optical Micromanipulation XV.

[112] Jean-Michel S. Market opportunities for advanced MEMS accelerometers and overview of actual capabilities vs. required specifications. Proceedings of the PLANS 2004. Position Location and Navigation Symposium (IEEE Cat. No. 04CH37556).

[113] Rafiee P., Khatibi G., Zehetbauer M. (2017). A review of the most important failure, reliability and nonlinearity aspects in the development of microelectromechanical systems (MEMS). Microelectron. Int..

[114] Clinton J.F., Heaton T.H. (2002). Potential advantages of a strong-motion velocity meter over a strong-motion accelerometer. Seismol. Res. Lett..

[115] Nag D., Chuan K.C.T. High performance ΣΔ closed loop accelerometer. Proceedings of the 2014 International Symposium on Integrated Circuits (ISIC).

[116] Aydemir A., Terzioglu Y., Akin T. (2016). A new design and a fabrication approach to realize a high performance three axes capacitive MEMS accelerometer. Sens. Actuators A Phys..

[117] Chamraz S., Balogh R. Analysis of capacitive MEMS sensor for small accelerations. Proceedings of the 2018 Cybernetics & Informatics (K&I).

[118] D’Emilia G., Gaspari A., Mazzoleni F., Natale E., Schiavi A. (2018). Calibration of tri-axial MEMS accelerometers in the low-frequency range—Part 1: Comparison among methods. J. Sens. Sens. Syst..

[119] Krishnamoorthy U., Olsson Iii R., Bogart G.R., Baker M., Carr D., Swiler T., Clews P. (2008). In-plane MEMS-based nano-g accelerometer with sub-wavelength optical resonant sensor. Sens. Actuators A Phys..

[120] He W.T., Chen D.Y., Li G.B., Wang J.B. (2012). Low frequency electrochemical accelerometer with low noise based on MEMS. Key Engineering Materials.

[121] Chen D., Li G., Wang J., Chen J., He W., Fan Y., Deng T., Wang P. (2013). A micro electrochemical seismic sensor based on MEMS technologies. Sens. Actuators A Phys..

[122] Deng T., Chen D., Wang J., Chen J., He W., Fan Y. A MEMS based electrochemical seismic sensor. Proceedings of the 2013 Transducers & Eurosensors XXVII: The 17th International Conference on Solid-State Sensors, Actuators and Microsystems (TRANSDUCERS & EUROSENSORS XXVII).

[123] Huang H., Agafonov V., Yu H. (2013). Molecular electric transducers as motion sensors: A review. Sensors.

[124] Chen L., Chen D., Wang J., Sun Z., Li G., Chen J. A MEMS based integrated three axial electrochemical seismic sensor. Proceedings of the 2017 19th International Conference on Solid-State Sensors, Actuators and Microsystems (TRANSDUCERS).

[125] Li G., Wang J., Chen D., Chen J., Chen L., Xu C. (2017). An Electrochemical, Low-Frequency Seismic Micro-Sensor Based on MEMS with a Force-Balanced Feedback System. Sensors.

[126] Sun Z., Chen D., Chen J., Deng T., Li G., Xu C., Wang J. (2016). A MEMS based electrochemical seismometer with low cost and wide working bandwidth. Procedia Eng..

[127] Chen D., Sun Z., Chen L., Li G., Wang J., Chen J. Numerical study of the frequency charateristics of the electrochemical seismometer. Proceedings of the 2017 IEEE SENSORS.

[128] Hortschitz W., Kainz A., Kovacs G., Steiner H., Stifter M., Sauter T., Schalko J., Jachimowicz A., Keplinger F. Robust, ultra sensitive MOEMS inertial sensor read out with infrared light. Proceedings of the 2018 IEEE Micro Electro Mechanical Systems (MEMS).

[129] Wang S., Wei X., Zhao Y., Jiang Z., Shen Y. (2018). A MEMS resonant accelerometer for low-frequency vibration detection. Sens. Actuators A Phys..

[130] U.S. Geological Survey (2017). Advanced National Seismic System—Current Status, Development Opportunities, and Priorities for 2017–2027.

[131] Rochford K., Strauss J., Kong Q., Allen R. (2018). MyShake: Using Human-Centered Design Methods to Promote Engagement in a Smartphonebased Global Seismic Network. Front. Earth Sci..

[132] Minson S.E., Brooks B.A., Glennie C.L., Murray J.R., Langbein J.O., Owen S.E., Heaton T.H., Iannucci R.A., Hauser D.L. (2015). Crowdsourced earthquake early warning. Sci. Adv..

[133] Alavi A.H., Buttlar W.G. (2019). An overview of smartphone technology for citizen-centered, real-time and scalable civil infrastructure monitoring. Future Gener. Comput. Syst..

[134] Kong Q., Lv Q., Allen R.M. Earthquake Early Warning and Beyond: Systems Challenges in Smartphone-based Seismic Network. Proceedings of the 20th International Workshop on Mobile Computing Systems and Applications.

